# Fellow-Workers and the Roll of Honour

**Published:** 1915-11-13

**Authors:** 


### ROUND THE WORLD.

[The Editor invites Early Information and Contributions Marked "Fellow-Workers."]

### Fellow-Workers and the Roll of Honour.

FURTHER deeds of heroism by members of the R.A.M.C. are recorded in a Special Supplement to the *London Gazette* which has been published since our last issue went to press. In recognition of their conspicuous gallantry and devotion to duty, six practitioners have been appointed to be Companions of the Distinguished Service Order, and eight have had conferred on them the Military Cross. These distinctions have been awarded for conduct during the last advance on the Western front.

MAJOR G. WALLACE G. HUGHES, M.R.C.S., L.R.C.P., 6th Cavalry Field Ambulance, has won the D.S.O. for work in arranging for the care of the wounded at Loos while that place was under heavy bombardment.

CAPTAIN W. J. E. BELL, M.B., No. 2 Field Ambulance, has received his appointment in the same Order for devotion to duty on all occasions, notably near Loos between September 28 and October 1, when he visited the advanced bearer post day and night under continuous shell fire, and personally supervised the arrangements for collecting and evacuating the wounded in that area.

CAPTAIN (TEMPORARY MAJOR) J. W. BIRD, M.R.C.S., L.R.C.P., 6th London Field Ambulance, R.A.M.C. (T.), gained his D.S.O. for his conduct during operations at Maroc and Loos. It is recorded that "on one occasion he worked for twenty-three hours without any cessation in dressing and tending the wounded. He set a fine example, which had far-reaching results."

TEMPORARY CAPTAIN C. S. P. HAMILTON, M.R.C.S., L.R.C.P., R.A.M.C., attached 2nd Battalion, The Buffs (East Kent Regiment), has been appointed to the Order for conspicuous gallantry in France. He dressed the wounded in the firing line, being for hours together under heavy shell fire, and went to points of great danger, often to where bombers were actually fighting.

CAPTAIN F. R. KERR, M.B., R.A.M.C., has won the D.S.O. for gallantry at Cuinchy. It is recorded that "after an unsuccessful attack on the enemy's trenches this officer crawled over our parapet and brought in a wounded man from about a dozen yards outside in full view of the enemy at a range of only seventy yards. He then went out again for thirty yards and rescued a man whose thigh had been broken, being fired at the whole time. During the night of September 25 Captain Kerr was out attending to the wounded for two hours under constant machine-gun and rifle fire, and on the night of September 27-28 he went to within twenty-five yards of the enemy's position to rescue a man reported wounded, but found that he was dead."

CAPTAIN A. J. A. MENZIES, M.B., R.A.M.C., attached 1st (Royal) Dragoons, has been appointed to the Distinguished Service Order for the duties he fulfilled with such heroism in Loos. He was twice seen carrying wounded on a stretcher under rifle fire, and for fifty-five hours he was continually exposing himself to heavy shell fire while carrying out his duties.

CAPTAIN F. P. FREEMAN, L.A.H. (Dublin), R.A.M.C. (Special Reserve), attached 23rd Field Ambulance, has had conferred upon him the Military Cross for his exploits during operations near Hulluch. He brought in and attended to the wounded during four consecutive days and nights, repeatedly going out under heavy fire. He set a splendid example to his men.

CAPTAIN J. R. MCCURDIE, M.B., R.A.M.C. (Special Reserve), attached to No. 2 Field Ambulance, has been awarded the same distinction for his gallantry at Le Rutoire farm, where, although continuously exposed to shell fire, he collected and treated the wounded. By his efforts a large number of wounded were collected.

TEMPORARY CAPTAIN J. M. MCLAGGAN, M.B., R.A.M.C., attached 3rd Battalion the Royal Fusiliers (City of London Regiment), receives the Military Cross for attending to the wounded in the firing line under heavy shell and rifle fire. His coolness and skill undoubtedly saved many lives. For three days and four nights he worked incessantly with unflagging energy.

TEMPORARY CAPTAIN C. J. O'REILLY, M.D., 21st Field Ambulance, is awarded the Military Cross for deeds performed during operations near Hulluch. He brought in wounded for four consecutive days and nights under heavy fire, and on one occasion he voluntarily went out to collect wounded under very heavy shell fire. He has consistently set a splendid example to his men.

CAPTAIN T. WALKER, M.B., R.A.M.C. (Special Reserve), attached No. 2 Field Ambulance, receives the same distinction for collecting wounded from an area under continuous shell fire, and at first under machine-gun fire also.

TEMPORARY LIEUTENANT D. C. ALEXANDER, M.B., R.A.M.C., attached to 5th Battalion the Queen's Own Cameron Highlanders, has had conferred on him the Military Cross for attending to wounded men who were lying in the open under enfilade machine-gun fire. On several occasions he carried out his duties under heavy shell fire.

TEMPORARY LIEUTENANT J. B. BAIRD, M.B., No. 1 Field Ambulance, when in charge of different bearer sections, collected wounded in the area between Lone Tree and Hulluch Road under shell and machine-gun fire. For this he has had conferred on him the Military Medal.

TEMPORARY LIEUTENANT G. RANKINE, M.B., R.A.M.C., attached Headquarters, 9th Divisional Royal Engineers, has been the recipient of the same honour. It is recorded that on one occasion he went with a party of bearers as far as Hohenzollern Redoubt, and, in spite of shell fire and bombing, assisted to get back many wounded. On the return journey many of the bearers were killed and wounded by a shell, and Lieutenant Rankine carried in a wounded man on his back.

TEMPORARY LIEUTENANT B. S. BROWNE, M.B., R.A.M.C., attached 2nd Battalion, the Cheshire Regiment, spent the whole night of October 2 searching for and carrying back wounded who were lying between our own and the enemy's lines, which were only 200 yards apart. After daybreak he carried back three more men under a very heavy fire. At one time he tended the wounded within fifteen yards of the enemy's trenches. By his courage all the wounded in his area were brought in. He has been awarded the Military Cross.

(*Continued on p.* 146.)

### Round the World.

(*Continued from p.* 142.)

Surgeon-General Sir Arthur Thomas Sloggett, K.C.B., C.M.G., has had conferred upon him by the President of the French Republic the decoration of the Legion of Honour for distinguished service in the field.

Colonel the Honourable Joseph Livesley Beeston, of the Australian Army Medical Corps, has been appointed to the Order of St. Michael and St. George for distinguished service in the field during the operations at the Dardanelles.

Lieutenant A. L. E. F. Coleman, M.D., R.A.M.C., attached to the 11th Middlesex Regiment, is included in the list of officers wounded published on November 5.

Captain J. E. Stacey, M.B., R.A.M.C., attached to the 5th Berkshire Regiment, is reported wounded. Captain Stacey was formerly house surgeon at the Jessop Hospital for Women, Sheffield.

